# *In Vitro* Antibacterial and Antibiotic Resistance Modifying Effect of Bioactive Plant Extracts on Methicillin-Resistant *Staphylococcus epidermidis*

**DOI:** 10.1155/2013/760969

**Published:** 2013-10-10

**Authors:** Romana Chovanová, Mária Mikulášová, Štefánia Vaverková

**Affiliations:** ^1^Department of Molecular Biology, Faculty of Natural Sciences, Comenius University, Mlynská Dolina, 842 15 Bratislava, Slovakia; ^2^Department of Pharmacognosy, Faculty of Pharmacy, Comenius University, Odbojárov 10, 832 32 Bratislava, Slovakia

## Abstract

The crude extracts of plants from Asteraceae and Lamiaceae family and essential oils from *Salvia officinalis* and *Salvia sclarea* were studied for their antibacterial as well as antibiotic resistance modifying activity. Using disc diffusion and broth microdilution assays we determined higher antibacterial effect of three *Salvia* spp. and by evaluating the leakage of 260 nm absorbing material we detected effect of extracts and, namely, of essential oils on the disruption of cytoplasmic membrane. The evaluation of *in vitro* interactions between plant extracts and oxacillin described in terms of fractional inhibitory concentration (FIC) indices revealed synergistic or additive effects of plant extracts and clearly synergistic effects of essential oil from *Salvia officinalis* with oxacillin in methicillin-resistant *Staphylococcus epidermidis*.

## 1. Introduction

*Staphylococcus epidermidis* belongs to the group of coagulase negative staphylococci (CoNS). It is commensal microorganism that constitutes a major component of the normal skin and mucosal microflora of humans [1]. However, in recent years these bacteria have emerged as an opportunistic pathogen, important causative agents of bacteremia, and the leading cause of nosocomial infections particularly associated with indwelling medical devices (e.g., prosthetic joints and heart valves) and in individuals with a compromised immune system (e.g., cancer patients and neonates) [2, 3]. *Staphylococcus epidermidis *pathogenesis relies on the ability to adhere and form biofilms on the surfaces of the medical devices mentioned earlier [4].

Staphylococci are considered as naturally susceptible to almost all antimicrobial agents developed but at the same time have a reputation of rapidly developing resistance [5]. CoNS, especially *S. epidermidis, *are often multiresistant, including resistance to methicillin. Resistance to methicillin is at 75–90% among hospital isolates of *S. epidermidis*, which is even higher than the corresponding rate for *S. aureus *(40–60%) [6]. In addition to methicillin resistance, *S. epidermidis *strains have acquired resistance to several other antibiotics. Most antibiotic resistance genes are plasmid-encoded and are more often found in methicillin-resistant than methicillin-susceptible strains [7]. These facts together with the ubiquity of *S. epidermidis *as a human commensal microorganism render this bacterium an optimal carrier and reservoir for antibiotic resistance genes and for the transfer of genetic elements to pathogenic bacteria.

The increasingly growing rate of antibiotic resistance of microorganisms necessitates the development and research of new antimicrobial agents or resistance modifiers. Medicinal plant-derived compounds have increased widespread interest in the search of alternative antibacterial agents because of the perception that they are safe and have a long history of use in folk medicine for the treatment of infectious diseases [8]. Natural products of higher plants may possess a new source of antimicrobial agents with possibly novel mechanisms of action [9, 10]. They are effective in the treatment of infectious diseases while simultaneously mitigating many of the side effects that are often associated with conventional antimicrobials [11]. Systematic and methodical screening of them may result in the discovery of novel active compounds [12].

Reversal of multidrug resistance may be another attempt to mitigate the spread of resistance. One of the promising methods in coping with bacterial resistance is, along the use of alternative classes of antimicrobial agents, also the application of synergistic activity between antibiotics and nonantibiotics. Many plants have direct antimicrobial activity but also resistance modifying/modulating activities [13]. Resistance modifying agents may inhibit multidrug resistance mechanism. This ability of plant extracts to enhance antibiotics has not been well defined. 

It is well documented that some plants belonging to Asteraceae and Lamiaceae families possess suitable medicinal properties, which are based mainly on the presence of essential oils [14]. Antimicrobial activity of many species of *Salvia* plants against several microorganisms has been recognized for decades and has been attributed to the presence of 1,8-cineole, *β*-thujone, camphor, borneol, and p-cymene, among others [15]. Terpenes of essential oils extracted from different herbs are proved to have antimicrobial activity and some of them may act as resistance modifiers.

Researchers studied antibacterial properties of various plants against Gram-negative as well as Gram-positive bacterial strains including *Staphylococcus aureus *[16–18], but there are only few reports on antibacterial activity against *Staphylococcus epidermidis*. Moreover, the interactions between plant extracts or essential oils and antibiotic in methicillin-resistant *S. epidermidis* have not been documented earlier.

The principal objective of the present study was to evaluate *in vitro *antibacterial activities of selected plant extracts from Asteraceae and Lamiaceae family against clinical isolates of *S. epidermidis *and to evaluate interactions between antibiotic and plant extracts or essential oils in coping with methicillin resistance.

## 2. Materials and Methods

### 2.1. Plant Extracts

In this work crude extracts of plants from Asteraceae (*Anthemis tinctorium, Chamaemelum nobile, Matricaria recutita, Tanacetum argyrophyllum,* and *Tanacetum parthenium*) and Lamiaceae (*Salvia fruticosa, Salvia officinalis,* and *Salvia sclarea*) family were used. The plants were harvested at the optimal growing and development stage. Essential oils from *Salvia officinalis* and *Salvia sclarea* were prepared in accordance with the European Pharmacopoeia [19]. Air-dried aerial parts were subject to hydrodistillation for 4 h, and isolated oil was diluted in n-hexane and dried over anhydrous sodium sulphate. 

### 2.2. Bacterial Strains

Methicillin-resistant and methicillin-susceptible *Staphylococcus epidermidis* strains were isolated from patients with positive haemocultures from University Teaching Hospital Old Town, Bratislava, Slovak Republic, and were kindly provided by Dr. Slobodníková from Institute of Microbiology, Faculty of Medicine, Comenius University in Bratislava, Slovak Republic. Strains are marked as Sep1 to Sep7. All strains were routinely grown aerobically in brain-heart infusion medium (Biomark, India) with shaking for 24 h at 37°C. 

### 2.3. Disc Diffusion Assay

The plant extracts were tested for antimicrobial activity by disc diffusion assay on Mueller-Hinton agar (Himedia, India). Suspension of the tested bacteria (0.1 mL of 10^8^ cells/mL) was spread onto solid media plates. The sterile paper discs (6 mm in diameter), which were impregnated with 10 *μ*L of individual extract, were placed on the incubated plates. These plates after 2 h of maintenance at 4°C were incubated for 24 h at 37°C and the diameters of the resulting zones of inhibition were measured in millimeters. 

### 2.4. Determination of Minimum Inhibitory Concentration (MIC)

The MIC values of plant extracts were determined by broth microdilution method using 96-well microtiter plates in accordance with CLSI (2011) guidelines [20]. Serial twofold dilutions of the plant extracts were prepared by vortexing the extracts in Millipore water. Inoculum of microorganism was prepared in Mueller-Hinton Broth (Himedia, India), and the turbidity was adjusted to 0.5 McFarland and diluted to obtain a final turbidity in wells approximately 1 × 10^6^ CFU/mL. Twenty *μ*L of solution of plant extract and 180 *μ*L of bacterial inoculum were placed into wells of microtiter plate and incubated at 37°C for 24 h. Growth of the bacteria was examined as a function of turbidity (optical density (OD) at 600 nm) using Varioskan Flash (Thermo Fisher Scientific, Finland). The MIC is defined as the lowest concentration of antimicrobial agent that completely inhibits growth of the organism.

### 2.5. PCR for *mec*A Gene

Detection of the *mec*A gene in *Staphylococcus epidermidis* strains was accomplished using polymerase chain reaction (PCR) amplification. Cells were suspended in a lysis buffer containing 1 M Tris HCl, 5 M NaCl, and 0.1 M EDTA, which was incubated at 95°C for 10 minutes. After incubation, the suspension was centrifugated at 23 000 ×g for 5 min. The supernatant was used as a template in PCR. PCR assay was carried out as described by Zhang et al. [21] using primers MecA147-F (GTGAAGATATACCAAGTGATT) and MecA147-F (GTGAAGATATACCAAGTGATT). The final PCR products were visualized using UV-transilluminator after electrophoresis on 1.5% agarose gel containing 50 mg/mL EtBr.

### 2.6. Checkerboard Method

The initial inoculum was prepared as described above. The 96-well microtiter plates were inoculated with test organism and serial dilutions of two antimicrobial agents—antibiotic and plant extract. Each well contained unique combination of plant extract/antibiotic concentrations. The plates were incubated for 24 h at 37°C. The absorbance of the plates was recorded at 600 nm using Varioskan Flash (Thermo Fisher Scientific, Finland).

Interactions between antimicrobial agents were determined by calculating the fractional inhibitory concentration (FIC) indices. The FIC is defined as follows: MIC of substance A tested in combination/MIC of substance A tested alone + MIC of substance B tested in combination/MIC of substance B tested alone. The FIC index is interpreted as FIC < 0.5—synergistic effect, 0.5 < FIC < 1—additive effect, 1 < FIC < 4—indifferent effect, and FIC > 4.0—antagonistic effect [22]. 

### 2.7. Time-Kill Assay

The effect of combinations of plant extract and oxacillin against methicillin-resistant *S. epidermidis* was evaluated using the time-kill assay method. All antimicrobial agents alone and in combination were tested against six strains of methicillin-resistant *S. epidermidis*. The concentration of extracts and oxacillin alone or in combination was 1/2 MIC. Time-kill curves were performed in tube containing nutrient broth, using inoculum density of approximately (10^7^ CFU/mL) in the presence of a single agent or a combination of antimicrobial agents. The tubes were continuously shaken and incubated at 37°C. Samples were obtained at 0, 6, 10, and 24 h. At each sample time, aliquots were taken and serially diluted. Fifty microliters of undiluted and diluted samples were plated on nutrient agar. The plates were incubated at 37°C for 24 h. After incubation, the numbers of colonies were enumerated and the mean counts (CFU/mL) for each test and controls were determined and expressed as log_10_. The effect of the antimicrobial combinations was interpreted as follows: synergy was defined as a decrease of ≥2 log_10_ CFU/mL in colony counts after 24 h by the combination compared to the most active single agent. Additivity or indifference was defined as a <2 log_10_ CFU/mL change in the average viable counts after 24 h for the combination, compared with the most active single agent. Antagonism was described as a ≥2 log_10_ CFU/mL increase in colony counts after 24 h by the combination compared to that by the most active agent alone [23]. 

### 2.8. Loss of 260 nm Absorbing Material

Loss of 260 nm absorbing material released from bacteria was measured by the technique of Devi et al. [24]. Bacterial suspension was prepared from overnight culture (OD_600_ 2.0). Cells were separated from medium by centrifugation at 400 ×g, for 15 min, washed twice in phosphate-buffered-saline (pH 7.4), and resuspended in the same buffer. Different concentrations of plant extracts 10%–0.03% (v/v) were added to the cell suspension. Ciprofloxacin (500 mg/L) and cell suspension without plant extract were used as controls. The samples were incubated at 37°C for 60 min with shaking. Samples from 0 and 60 min of the experiment were centrifuged at 13 400 ×g, for 15 min. For each time point and treatment agent optical density was measured at 260 nm with spectrophotometer (NanoDrop, Thermoscientific, USA). 

## 3. Results and Discussion

### 3.1. Antibacterial Effect

Six clinical isolates of methicillin-resistant *Staphylococcus epidermidis* (Sep1–Sep6) and one methicillin-susceptible strain (Sep7) were used to evaluate the possible antistaphylococcal activity of selected plant extracts. Our results, determined using disc diffusion and broth microdilution methods, are presented as average values in Tables 1 and 2. According to inhibition zone diameters, most effective were extracts from three species of *Salvia *(inhibition zone from 12.4 mm to 12.7 mm) followed by extract from *Matricaria recutita. *Extracts from the rest of *Asteraceae* family plants showed smaller inhibition zones, from 7.0 mm to 10.4 mm. There was no difference between inhibition zones of methicillin-resistant and methicillin-susceptible strains.

More precise data on the antimicrobial properties were obtained through the determination of minimum inhibitory concentration and the results from microdilution method confirmed the previous results. Extracts from* S. officinalis and S. sclarea *showed higher inhibitory properties with MIC values in the range of 1.25% to 10% (v/v) than those of *Asteraceae* extracts, most of which had MIC 10% or more than 10% (v/v).

Antibacterial effects of crude extracts and, namely, of essential oils from different species of *Salvia* on the growth of Gram-positive and Gram-negative bacteria were evaluated by many authors. Some of them confirmed antibacterial effect of different *Salvia *species also on staphylococci [25, 26], while other stated that extracts from *Salvia officinalis *[27], *S. divinorum *[28], and others had no effect on the growth of *Staphylococcus aureus *and *S. epidermidis.* However the compounds of essential oil from *S. sclarea*—abietane diterpenoids are bactericid for the cultures of *S. aureus *and *S. epidermidis *strains [29].

### 3.2. Loss of 260 nm Absorbing Material

**Figure 1:**
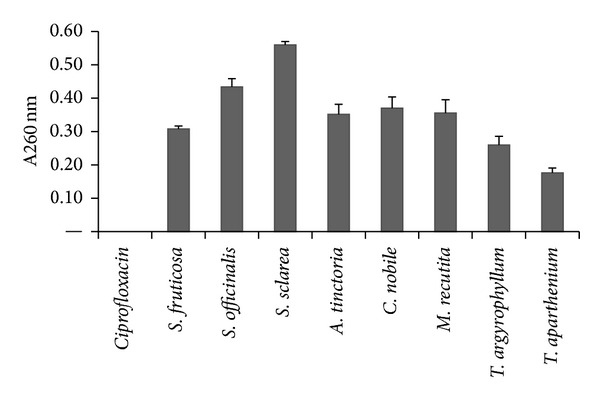
The appearance of 260 nm-absorbing material in the filtrates of *S. epidermidis *Sep6 after 60 min treatment with plant extracts in concentration of 5%. Ciprofloxacin was used as control. The means ± SD for at least three replicates are illustrated.

**Figure 2:**
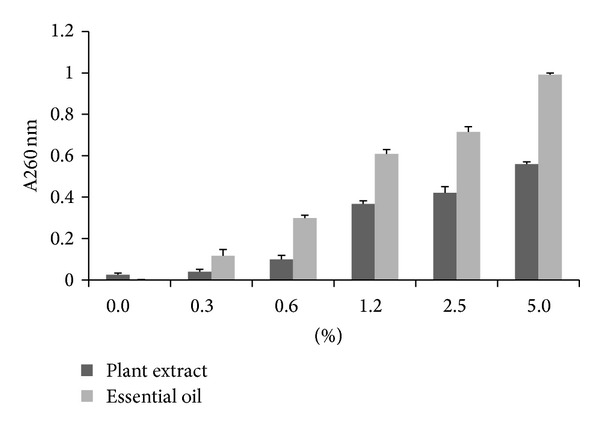
The effect of crude extract and essential oil from *Salvia sclarea *on the leakage of 260 nm absorbing materials from *Staphylococcus epidermidis *Sep6.

We extended our investigation of antibacterial effect of plant extracts to evaluation of its possible mechanism. The mechanism of action of terpenes from essential oils, which are main parts of tested plants, is not fully understood, but it is assumed that membrane perturbation by these lipophilic components is involved in the antibacterial action. Marked leakage of cytoplasmic material is considered indicative of gross and irreversible damage to the cytoplasmic membrane [30, 31] and is commonly quantified by the loss of intracellular material that absorb at wavelengths of 260 nm (nucleic acids). After treatment with plant extracts at increased concentrations from 0.3% to 5% (v/v) the OD_260_ of filtrates of all tested strains increased and the most remarkable increases occurred after 60 min treatment with 5% *Salvia sclarea* and 5%* Salvia officinalis *(Figure 1). At the same time the OD_260_ of control without extract and of control with ciprofloxacin were not changed. These results suggest that crude extracts from tested plants damage the cytoplasmic membrane and cause loss of intracellular components. In order to confirm the assumptions that this effect have been based on the nature of essential oils, in Figure 2 we compared the leakage of intracellular compounds in the presence of crude plant extract and of essential oil from the same plant *Salvia sclarea*. The effect of essential oil is significantly higher yet in lower concentrations. 

It has been reported that some antimicrobial agents cause gross membrane damage and provoke whole cell lysis [32]. Among these compounds can be found also essential oils from oregano, rosewood, and thyme [33], *α*-pinene [34], lemongrass oil [35], and tea tree oil from *Melaleuca alternifolia *[36].

### 3.3. Interactions between Plant Extracts and Oxacillin

**Figure 3:**
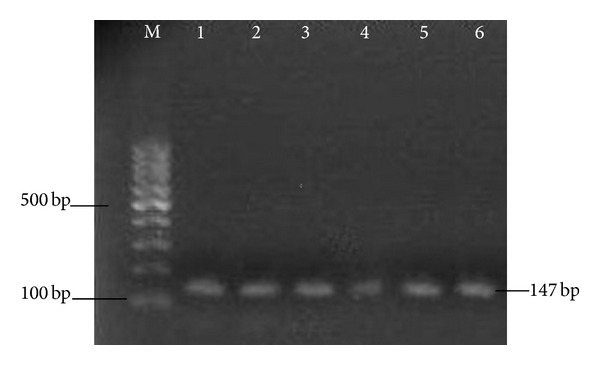
Detection of *mec*A gene. Lanes: M: 100 bp marker, 1–6: 147 bp PCR product of *mec*A gene from strains *Staphylococcus epidermidis* Sep1–Sep6.

The resistance of the six clinical strains *S. epidermidis *to oxacillin is obvious from the Table 2. The breakpoint for oxacillin resistance for CoNS according to Eucast 2013 [37] is MIC > 0.25 mg/L. MIC of oxacillin of our resistant strains (Sep1–Sep6) ranged from 16 mg/L to 256 mg/L, while MIC of methicillin-susceptible strain Sep7 was 0.125 mg/L. Methicillin resistance of Sep1–Sep6 strains was confirmed by determination of gene *mec*A (Figure 3). PCR for the *mec*A gene coding for methicillin resistance via penicillin binding protein 2a (PBP2a) is well established and is considered as “gold standard” for detection of methicillin resistance in comparison with phenotypic methods [38–40].

The evaluation of *in vitro* interactions between plant extracts and oxacillin are described in terms of fractional inhibitory concentration (FIC) indices. The FICs for all used plant extracts and oxacillin against bacterial strain of methicillin-resistant *S. epidermidis *Sep6 are shown in Table 3. Extracts from all three *Salvia* species combined with oxacillin had synergistic effect. From all *Asteraceae *only extract from *Matricaria recutita* had synergistic effect with oxacillin, and all rest extracts had additive effect. It was found that the crude extract of *Salvia officinalis *reduced the minimum inhibitory concentration of aminoglycosides in vancomycin-resistant *enterococci *and then the effective compound was isolated. Carnosol, the active compound showed weak antimicrobial activity and greatly reduced the MICs of various aminoglycosides [41]. The exact mechanism for the reduction of *β*-lactam (methicillin) resistance by the natural antimicrobials is unknown but is likely due to some structural change in the resistant bacteria. Epigallocatechin gallate from green tea inhibited the activity of penicillinase produced by *S. aureus *[42] and synergistically enhanced the activity of carbapenems against methicillin-resistant *S. aureus *(MRSA) [43]. Tellimagrandin I from red rose (*Rosa canina* L.) petal extract greatly reduced the MIC of *β*-lactam antibiotic against MRSA [44]. Similarly, corilagin, an active compound extracted from *Arctostaphylos uva-ursi,* was found to reduce the MICs of oxacillin and cefmetazole against MRSA [45]. 

**Figure 4:**
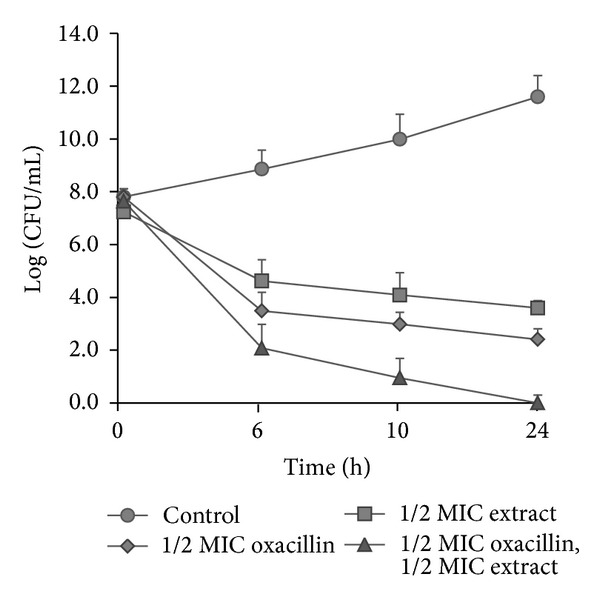
Time-kill curves of *S. epidermidis* Sep6 after treatment with 1/2 MIC of oxacillin, 1/2 MIC of extract from *Salvia officinalis* alone or in combination.

The synergistic effects arising from the combination of oxacillin and plant extract from *Salvia* species in checkerboard assays were explored in greater detail by using time-kill assays. As is shown in Figure 4, the synergistic effects (difference ≥2 Log_10_) could be observed starting from 10 hours of incubation and continued up to 24 hours. The results of the checkerboard and time-kill assays agreed in all cases. 

### 3.4. Interaction between Essential Oil from *Salvia officinalis *and Oxacillin

Regarding the synergistic effect of extracts from* Salvia* species, in this part of work we were concerned with evaluation of interactions between essential oils from *Salvia officinalis *and oxacillin. The results of the checkerboard method indicated synergism in all six strains of methicillin-resistant *S. epidermidis* for which the combination of essential oil and oxacillin were tested (Table 4). FICI 1.811 for methicillin-susceptible strain Sep7 indicated indifferent effect. This is in accordance with the fact that the combination of two agents exhibit significant synergism only if the test organism is resistant to at least one of the agents [46].

The observed synergistic effects of plant extracts (essential oils) and oxacillin could be theoretically the results of the perturbation of the cell membrane coupled with the action of oxacillin. *β*-lactam antibiotic oxacillin inhibits the final stage involved in the synthesis of peptidoglycan of cell wall (transpeptidation reaction), which occurs outside the cell membrane and is mediated by alternative protein binding protein PBP2a encoded by *mec*A gene [47]. 

Compounds having synergic effect with oxacillin may inhibit the PBP2a activity or inhibit its production [45]. First mechanism can be connected with the perturbation of cell membrane, which we have confirmed in this work; the second mechanism: the inhibition of the production PBP2 by inhibition of *mec*A gene expression is the content of our current research and its result will be published in later stage. 

## 4. Conclusion

The antibacterial activity of plant extracts from *Asteraceae *and* Lamiaceae* family was confirmed and contributed to the ability of contained essential oils to disturb biological membranes. Synergistic activity of extracts as well as essential oil from *S*. *officinalis *and oxacillin could suggest an alternative manner to overcome a problem of bacterial infections caused by methicillin resistant *Staphylococcus epidermidis*. Further research is necessary to identify active compounds and research mechanism of interaction with antibiotics.

## Figures and Tables

**Table 1:** Inhibition zone diameter in mm as established by disc diffusion method.

Plant extract	Zone of inhibition (mm)^a^							Mean
	Sep1	Sep2	Sep3	Sep4	Sep5	Sep6	Sep7	
*Salvia fruticosa *	13	10	13	10	13	16	14	12.7
*Salvia officinalis *	12	11	14	10	14	15	13	12.7
*Salvia sclarea *	12	9	11	12	13	15	15	12.4
*Anthemis tinctoria *	7	7	6	6	8	7	8	7.0
*Chamaemelum nobile *	10	9	10	10	9	11	10	9.8
*Matricaria recutita *	10	8	8	9	15	15	14	11.3
*Tanacetum argyrophyllum *	9	8	7	8	13	12	8	9.3
*Tanacetum parthenicum *	12	9	12	10	10	12	8	10.4

^a^Diameter includes 6 mm disc.

**Table 2:** Minimum inhibition concentration (MIC) of plant extracts and oxacillin established by both microdilution method. MIC is given as % (v/v) for plant extracts and mg/L for oxacillin.

Plant extract	Minimum inhibitory concentration						
	Sep1	Sep2	Sep3	Sep4	Sep5	Sep6	Sep7
*Salvia fruticosa *	10	10	10	10	10	10	10
*Salvia officinalis *	2.5	2.5	5	10	1.25	5	5
*Salvia sclarea *	10	1.25	10	10	5	10	10
*Anthemis tinctoria *	>10	>10	>10	>10	>10	>10	>10
*Chamaemelum nobile *	≥10	>10	≥10	≥10	>10	10	>10
*Matricaria recutita *	≥10	>10	>10	>10	5	5	10
*Tanacetum argyrophyllum *	>10	>10	>10	>10	5	10	>10
*Tanacetum parthenicum *	10	>10	10	≥10	≥10	10	>10
Oxacillin	128	32	16	256	32	32	0.125

**Table 3:** Interactions of plant extracts and oxacillin in effect on methicillin-resistant *S. epidermidis *Sep6.

Plant extract	FIC A	FIC B	FIC	Interpretation
*Salvia fruticosa *	0.03	0.17	0.20	Synergistic
*Salvia officinalis *	0.05	0.09	0.14	Synergistic
*Salvia sclarea *	0.06	0.09	0.15	Synergistic
*Anthemis tinctoria *	0.12	0.47	0.59	Additive
*Chamaemelum nobile *	0.13	0.41	0.54	Additive
*Matricaria recutita *	0.06	0.12	0.18	Synergistic
*Tanacetum argyrophyllum *	0.26	0.53	0.79	Additive
*Tanacetum parthenicum *	0.24	0.51	0.75	Additive

FIC A = MIC of substance A tested in combination/MIC of substance A tested alone, FIC B = MIC of substance B tested in combination/MIC of substance B tested alone. FIC = FIC A + FIC B. A—oxacillin, B—plant extract.

**Table 4:** Interactions of essential oil from *Salvia officinalis* with oxacillin in effect on clinical strains of methicillin-resistant *S. epidermidis*.

*S. epidermidis *	FIC A	FIC B	FIC	Interpretation
Sep1	0.031	0.125	0.156	Synergistic
Sep2	0.062	0.250	0.312	Synergistic
Sep3	0.062	0.125	0.187	Synergistic
Sep4	0.062	0.250	0.312	Synergistic
Sep5	0.031	0.250	0.28	Synergistic
Sep6	0.031	0.125	0.156	Synergistic
Sep7	0.562	1.250	1.812	Indifferent

FIC A = MIC of substance A tested in combination/MIC of substance A tested alone, FIC B = MIC of substance B tested in combination/MIC of substance B tested alone. FICI = FIC A + FIC B. A—oxacillin, B—essential oil.
